# Supplementary material for: Southern Africa crustal anisotropy reveals coupled crust-mantle evolution for over 2 billion years
Source: Nat Commun. 2019 Nov 29;10:5445. doi: 10.1038/s41467-019-13267-2 (PMC6884544; doi:10.1038/s41467-019-13267-2)
Supplement: Supplementary file 2 — Supplementary Information [file 41467_2019_13267_MOESM2_ESM.pdf]

Supplementary Information to

Southern Africa crustal anisotropy reveals coupled crust-mantle evolution for over 2 billion years

by H. Thybo et al.

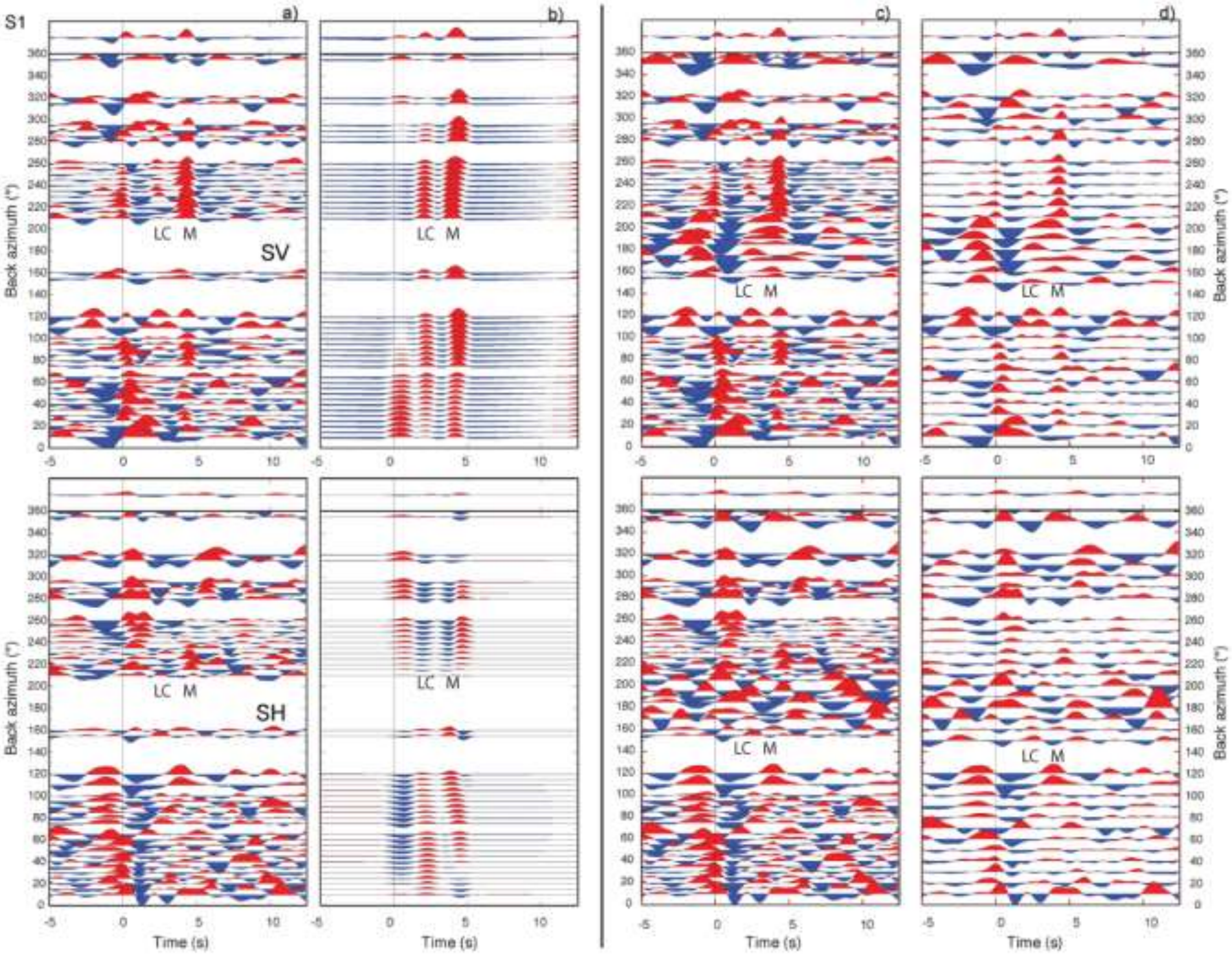

Supplementary Figure 1. Stacks of transverse P_{SH} and radial P_{SV} receiver functions (bandpass filtered to 1-20 s) versus back azimuth (stacked in 5° bins (a-c) and 10° bins (d)) for the western V2 unit[15] of Kaapvaal Craton together with synthetic receiver functions calculated for the models in Table 1. Upper panels show radial SV- and lower panels show transverse SH- receiver functions. a) RF excluding BAZ in the range between 165° and 205°, b) synthetic RFs for average model in Table S1, c and d) RFs for all BAZ ranges. LC – Top lower crust conversion, M – Moho conversion. The plots demonstrate that the observed features of the seismic data versus back-azimuth are robust within tectonic units.

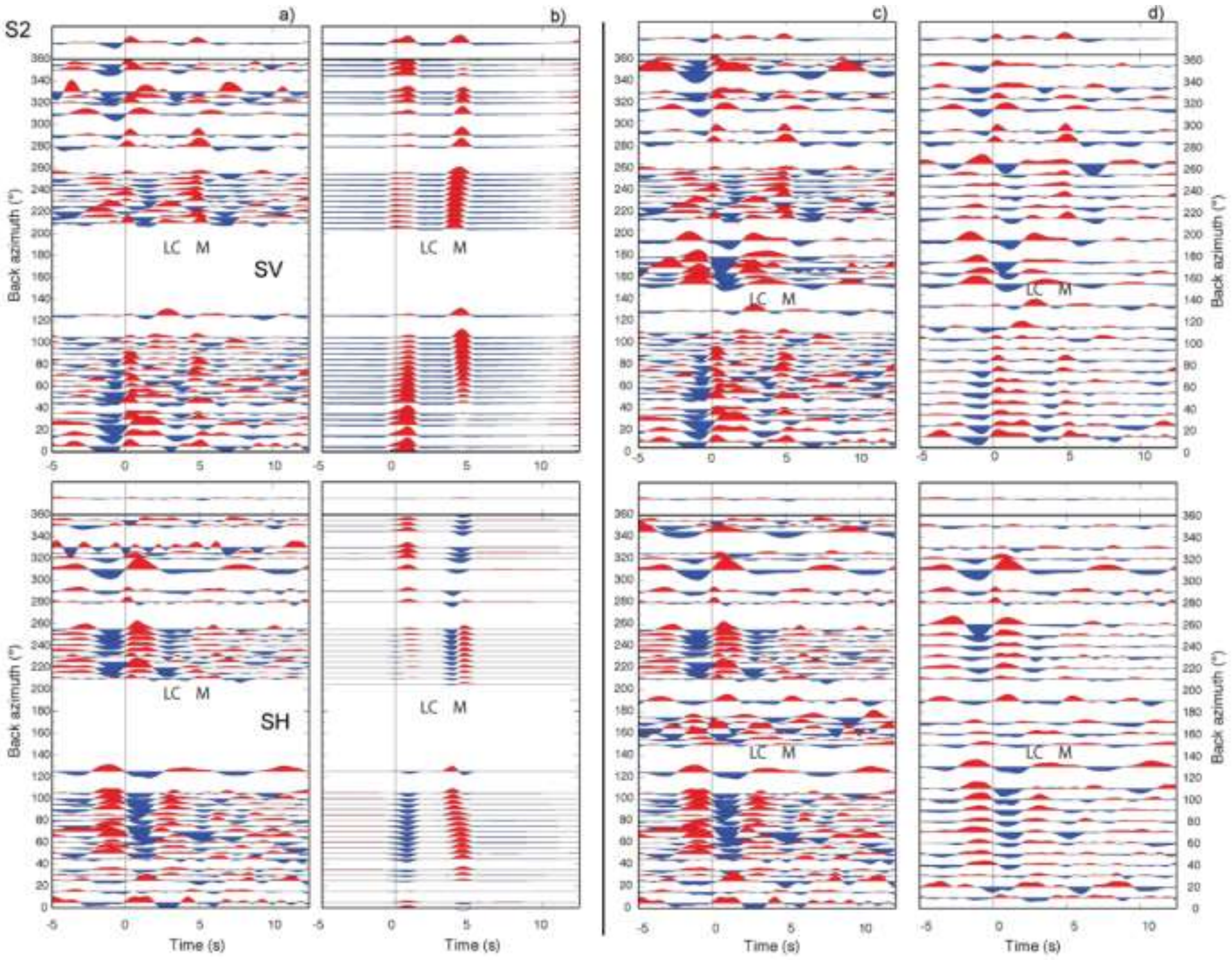

Supplementary Figure 2. Stacks of transverse P_{SH} and radial P_{SV} receiver functions (bandpass filtered to 1-20 s) versus back azimuth (stacked in 5° bins (a-c) and 10° bins (d)) for the eastern part of Limpopo Belt together with synthetic receiver functions calculated for the models in Table 1. Upper panels show radial SV- and lower panels show transverse SH- receiver functions. a) RF excluding BAZ in the range between 165° and 205°, b) synthetic RFs for average model in Table 1, c and d) RFs for all BAZ ranges. The plots demonstrate that the observed features of the seismic data versus back-azimuth are robust within tectonic units.

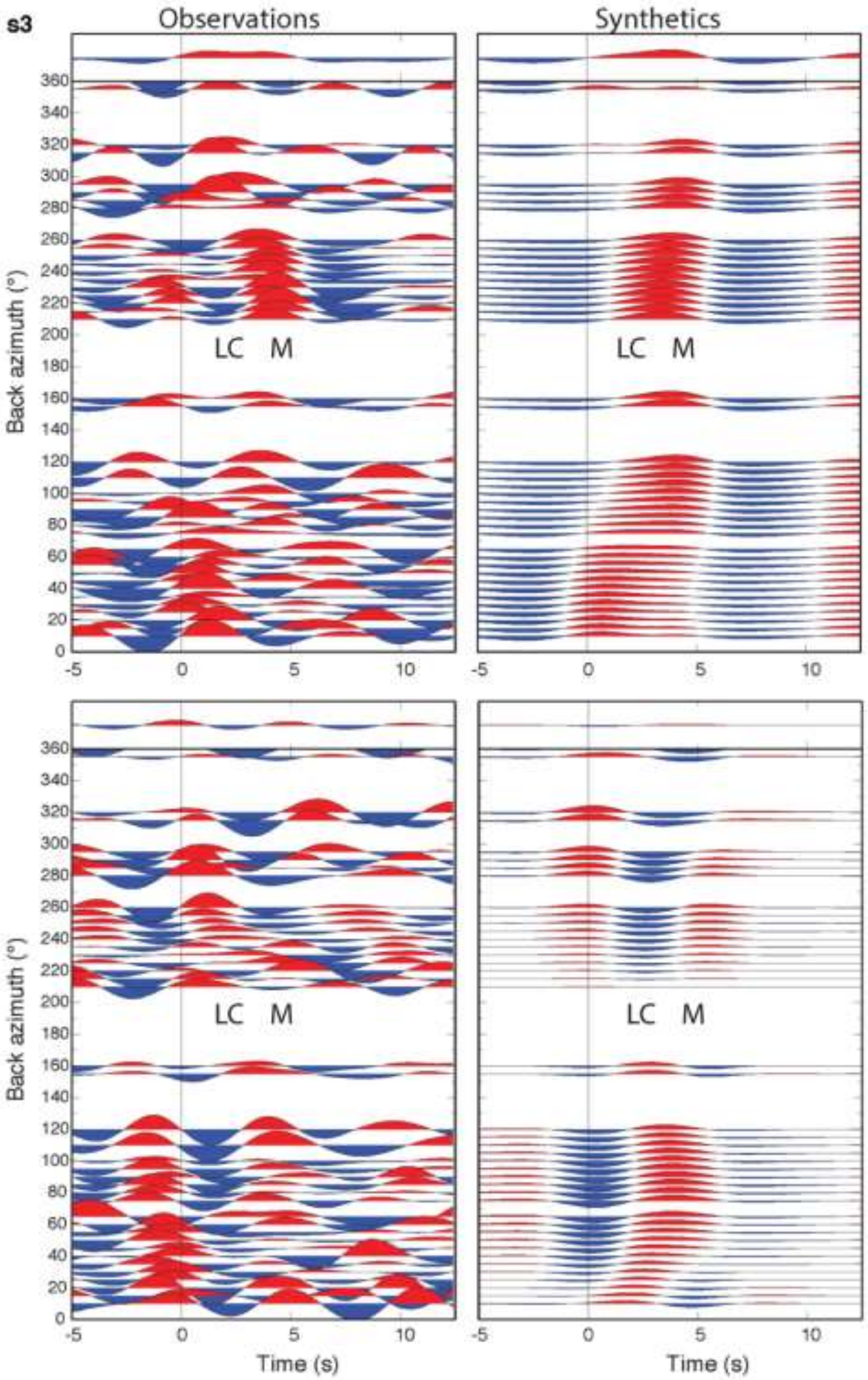

Supplementary Figure 3. Stacks of transverse P_{SH} and radial P_{SV} receiver functions (bandpass filtered to 5-20 s) versus back azimuth (stacked in 5° bins) for the western V2 unit[29] of Kaapvaal Craton together with synthetic receiver functions calculated for the models in Table 1.

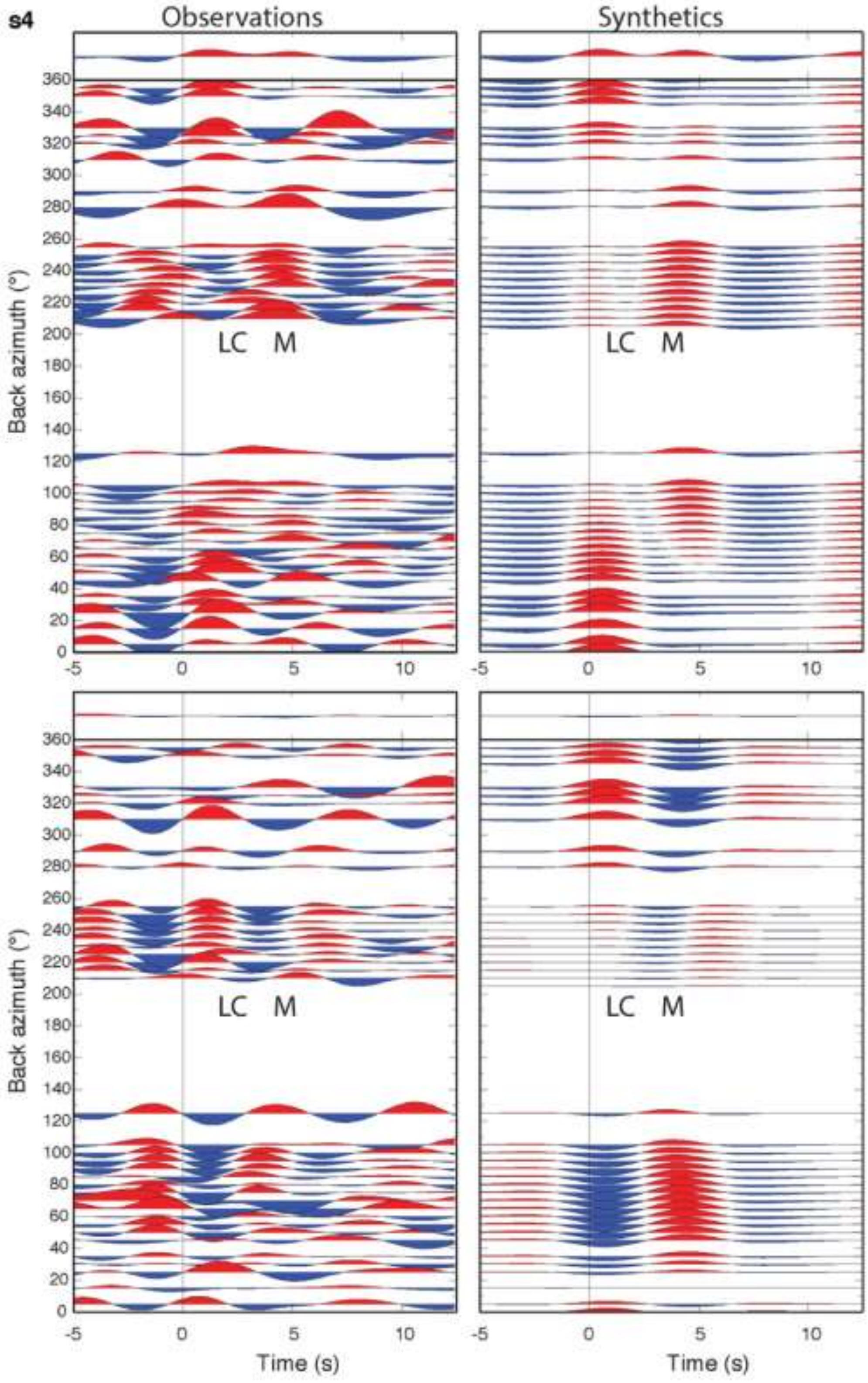

Supplementary Figure 4. Stacks of transverse P_{SH} and radial P_{SV} receiver functions (bandpass filtered to 5-20 s) versus back azimuth (stacked in 5° bins) for the eastern part of Limpopo Belt together with synthetic receiver functions calculated for the models in Table 1.

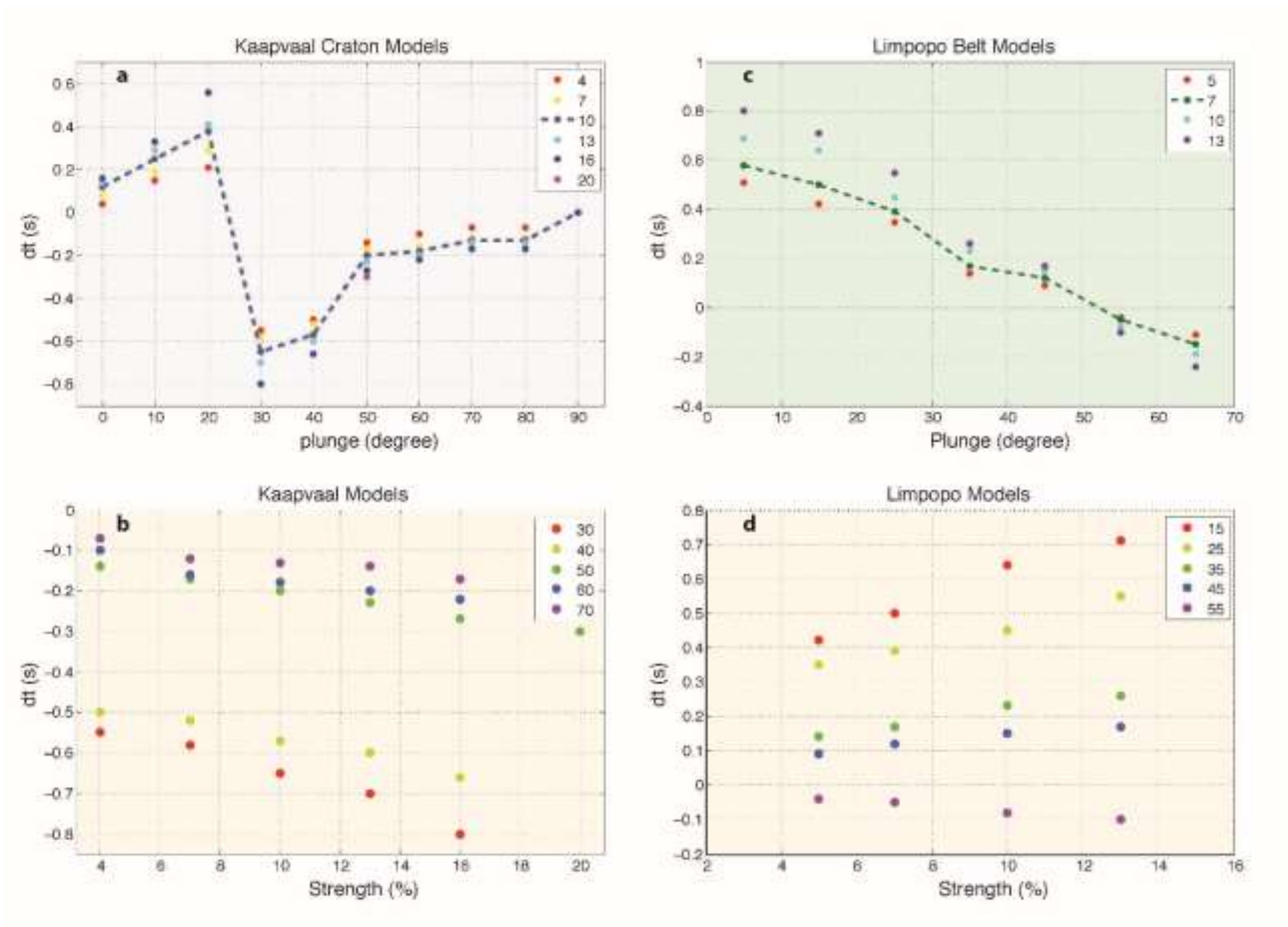

Supplementary Figure 5. Uncertainty estimation based on relative delay time (dt) between transverse S_H and radial S_V phases in Kaapvaal Craton and Limpopo Belt: a,c) dt versus plunge angle of fast direction in the lower crust for selected values of anisotropy strength in %; b,d) dt versus anisotropy strength in the lower crust for selected values of plunge angle in degrees. These plots indicate that the uncertainties of strength and plunge angles are ±4% and ±10°, respectively.

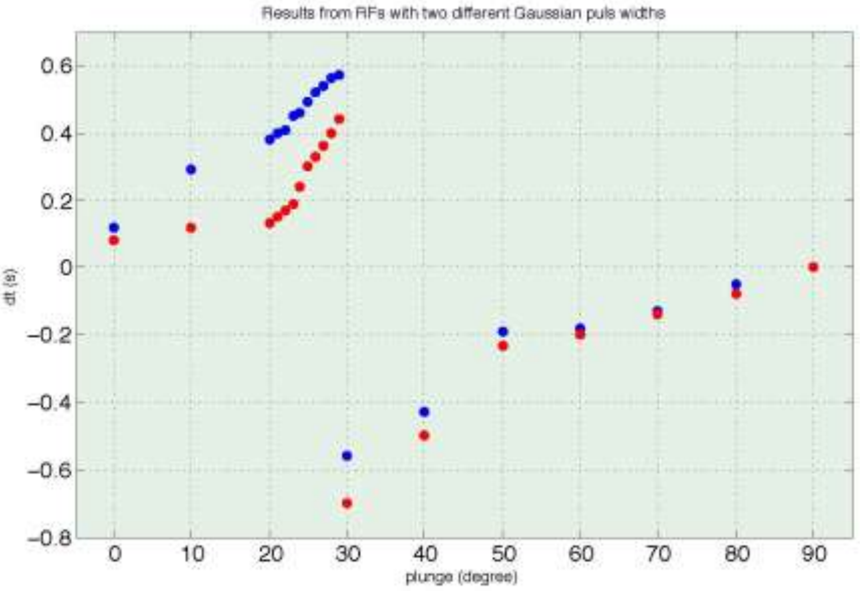

Supplementary Figure 6. Illustration of the polarity change versus plunge angle for the Kaapvaal model. The delay time between transverse S_H and radial S_V phases (dt) has been determined at two frequency bandwidths (at 0.75 s and 1.5 s periods) at a range of plunge angles. The polarity flip occurs at the same plunge angle of 30° for both frequencies, indicating that this is a feature related to the anisotropic structure of the crust and not an artefact from interference between the seismic waves.

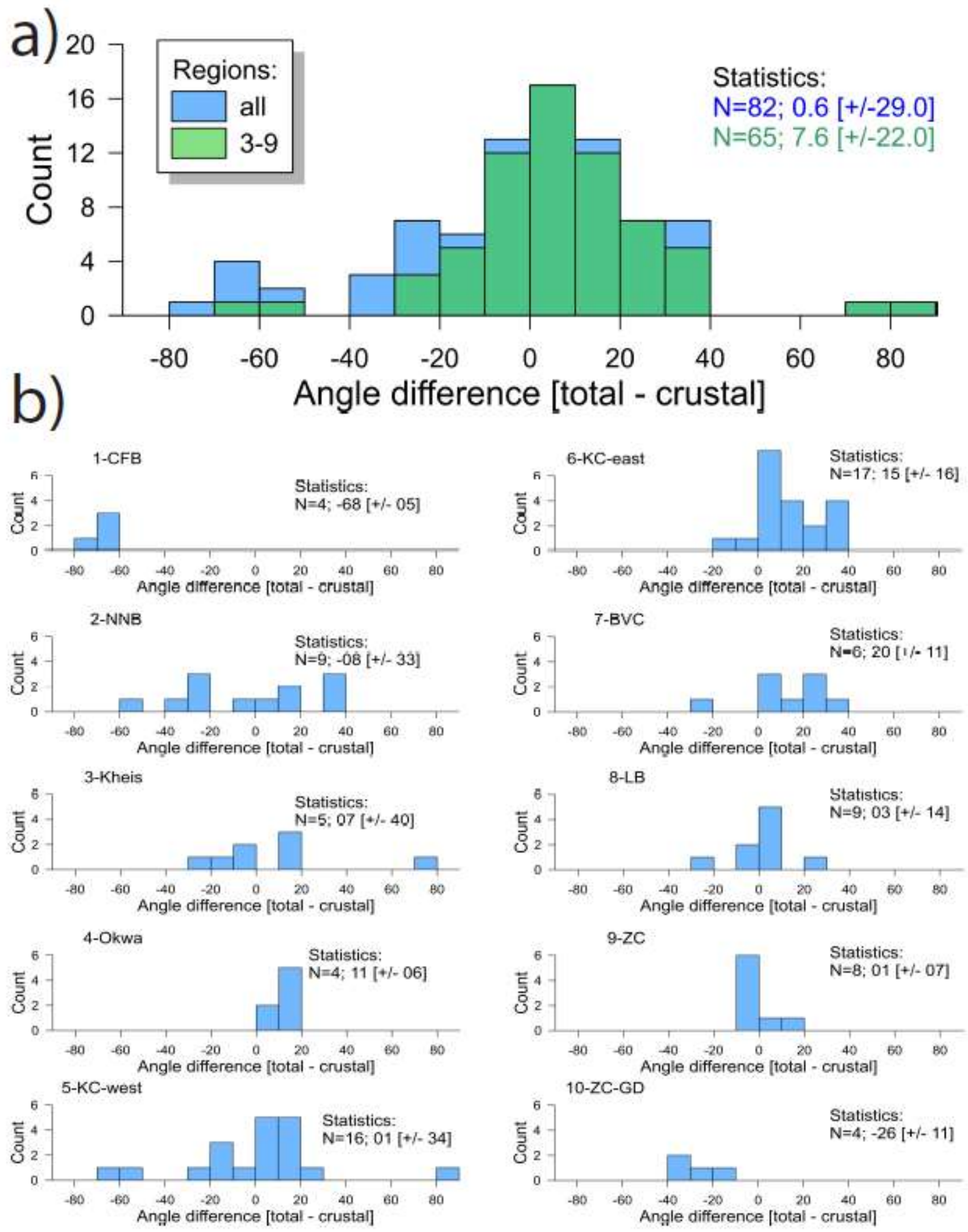

Supplementary Figure 7. Histograms showing difference between fast axis directions for SKS-splitting and receiver function interpretations. a) For all stations (blue) and for stations in areas 3-9 only (see below). **b)** For individual areas. Abbreviations: CFB – Cape Fold Belt, NNB - Namaqua-Natal Belt, Kheis – Belt, Okwa – Okwa Belt, KCw – West Kaapvaal Craton, KCe – East Kaapvaal

Craton, BVC – Bushveld Complex, LB – Limpopo Belt, ZC – Zimbabwe Craton, ZC_GD – Zimbabwe Craton around the great Dyke.

Supplementary Table

Station	Moho Depth (km)	Fast axis (degree)	δTc (s)	Category
**Zimbabwe**				
SA72	38	40	-0.2	A
SA75	37	48	+0.26	B
SA76	36	43	-0.15	A
SA78	36.5	45	na	D
SA79	35	35	-0.25	C
SA80	37	52	-0.21	C
**W Zimbabwe**				
SA66	46.5	65	-0.23	C
SA67	39.5	65	+0.18	C
SA70	50.5	60	+0.23	B
SA71	40.5	45	+0.18	C
**Limpopo Belt**				
SA55	44.5	68	+0.13	B
SA155/SA92	44.5	73	+0.1	D
SA56	42.5	60	+0.12	C
SA57	41.5	66	0	A
SA65	43	55	+0.17	C
SA68	41	60	+0.22	B
SA69	54.5	70	-0.12	D
SA169/SA93	43	68	+0.16	C
SA73	46	73	+0.15	C
SA74	34.5	78	+0.17	C
SA77	39.5	55	+0.25	B
**N. Kaapvaal (Bushveld complex, Barberton Greenstone belt, Pietersburg-Giyani-Murchison block)**				
SA42	36	43	+0.16	A
SA43	39	48	+0.21	A
SA44	44	50	-0.18	B
SA46	41.5	28	+0.19	C
SA47	49.5	40	-0.17	B
SA48	45.5	40	-0.17	B
SA49	48.5	38	-0.25	A
SA51	45	47	-0.21	B
SA52	39.5	45	-0.16	B
SA53	33.5	53	+0.17	B
SA54	37	52	-0.32	B
SA58	38.5	62	-0.38	C

Station	Moho Depth (km)	Fast axis (degree)	δTc (s)	Category
**NW Kaapvaal (Gaborone Granites)**				
SA45	46	33	+0.19	B
SA50	39	40	-0.17	C
SA59	41.5	37	+0.17	C
SA60	41.5	50	+0.19	C
SA62	40.5	42	-0.27	A
SA63	43	52	-0.18	A
**E. Kaapvaal (Witwatersrand Basin)**				
SA26	38.5	39	-0.14	B
SA32	39	38	+0.18	C
SA33	33.5	35	+0.18	C
SA34	38	36	+0.16	C
SA40	43.5	37	-0.19	C
**W. Kaapvaal (Ventersdorp LIP)**				
SA13	36.5	40	+0.25	C
SA14	33.5	39	+0.05	D
SA15	36.5	38	+0.04	D
SA18	36	33	+0.19	B
SA19	36.5	45	-0.14	B
SA24	38	28	-0.19	C
SA25	37.5	30	-0.12	B
SA30	36.5	25	+0.19	C
SA31	38.5	37	-0.19	C
SA37	34	28	-0.11	B
SA38	39.5	27	-0.23	A
SA39	41.5	38	-0.23	C
SA139/SA91	41	35	-0.23	B
**SE Kaapvaal**				
SA20	35	47	-0.1	B
SA27	39	32	-0.14	B
SA28	41	33	+0.19	B
SA35	39	33	+0.16	B
SA36	36.5	36	+0.16	C
**Kheis-Okwa Mobile Belt**				
SA16	40	28	-0.21	C
SA17	38.5	50	+0.23	C
SA22	48	30	na	D
SA23	41.5	32	0	A
SA29	35	28	0	B
SA61	43.5	47	-0.39	C
SA64	41	57	na	C

Station	Moho Depth (km)	Fast axis (degree)	δTc (s)	Category
**Namaqua-Natal Mobile Belt (NNMB)**				
SA05	41	65	+0.27	D
SA07	46.5	42	+0.33	D
SA08	41.5	25	na	D
SA09	46	22	+0.31	C
SA10	44.5	27	-0.27	C
SA11	42	25	-0.48	C
SA12	43	35	-0.34	C
SA81	45	41	0	A
SA82	48	45	0	A
**Cape Fold Belt (CFB)**				
SA01	33.5	67	0	A
SA02	47	68	-0.17	C
SA03	48	75	-0.53	B
SA04	34	70	na	D

Supplementary Table 1. Moho depth, fast axis direction (ϕ_c) and the amplitude (δt_c) of crustal anisotropy for each SASE station. Categories represent different data quality which are: A) excellent traces, B) good traces, in particular the Moho phases clear in SV component, C) moderate traces while it is very difficult to detect the inter-crustal discontinuities, D) complicated traces. The relation between the arrival time of the radial P_{SV} and the transverse P_{SH} RF is listed. δt_c is the difference in traveltime between the transverse SH and the radial SV arrivals.
